# Digestion in Ruminant Animals

**Published:** 1882-07

**Authors:** 


Digestion in Ruminant Animals.—We would call the attention of our readers to the full and careful description of digestion in ruminants given in this and the preceding number of the Journal. A very large number of the diseases of cattle are connected with the digestive organs. A full knowledge of the subject is of the highest importance to the veterinarian therefore.

We may supplement Mr. Slee's description with some recent observations made by Professor Ellenberger upon the *Nervous Supply of the Stomachs of Ruminants.* He states, as the result of his experiments:

1. That the pneumogastric is the motor nerve for the stomachs of all ruminants.

2. The stomachs have, however, local ganglia, by means of which movements are possible even after both pneumogastrics are cut.

3. The second stomach, or recticulum, contracts voluntarily and quickly, like striped muscle. Its muscular coat, however, is made up of smooth muscle fibres. The paunch has also only smooth muscle, yet its movements are much more energetic and rapid than is ordinarily observed with such muscle.

4. The psalter, or third stomach, moves and functions quite independently of the three other stomachs. It has a special nerve supply, consisting of ganglia in its walls, with nerves running to the different leaves.

5. The psalter is excited to motion by local irritation. The course of excito-motor fibres from the nerve-centres is not known.

